# Evolution of multi-drug resistant HCV clones from pre-existing resistant-associated variants during direct-acting antiviral therapy determined by third-generation sequencing

**DOI:** 10.1038/srep45605

**Published:** 2017-03-31

**Authors:** Haruhiko Takeda, Yoshihide Ueda, Tadashi Inuzuka, Yukitaka Yamashita, Yukio Osaki, Akihiro Nasu, Makoto Umeda, Ryo Takemura, Hiroshi Seno, Akihiro Sekine, Hiroyuki Marusawa

**Affiliations:** 1 Department of Gastroenterology and Hepatology, Graduate School of Medicine, Kyoto University, Kyoto, Japan; 2 Department of Gastroenterology and Hepatology, Japanese Red Cross Wakayama Medical Center, Wakayama, Japan; 3 Department of Gastroenterology and Hepatology, Osaka Red Cross Hospital, Osaka, Japan; 4 Department of Gastroenterology and Hepatology, Hyogo Prefectural Amagasaki General Medical Center, Hyogo, Japan; 5 Clinical Research Center, Chiba University Hospital, Chiba, Japan; 6 Center of Preventive Medical Sciences, Chiba University, Chiba, Japan; 7 Department of Genomic Medicine Omics Research Center, National Cerebral and Cardiovascular Center, Osaka, Japan

## Abstract

Resistance-associated variant (RAV) is one of the most significant clinical challenges in treating HCV-infected patients with direct-acting antivirals (DAAs). We investigated the viral dynamics in patients receiving DAAs using third-generation sequencing technology. Among 283 patients with genotype-1b HCV receiving daclatasvir + asunaprevir (DCV/ASV), 32 (11.3%) failed to achieve sustained virological response (SVR). Conventional ultra-deep sequencing of HCV genome was performed in 104 patients (32 non-SVR, 72 SVR), and detected representative RAVs in all non-SVR patients at baseline, including Y93H in 28 (87.5%). Long contiguous sequences spanning NS3 to NS5A regions of each viral clone in 12 sera from 6 representative non-SVR patients were determined by third-generation sequencing, and showed the concurrent presence of several synonymous mutations linked to resistance-associated substitutions in a subpopulation of pre-existing RAVs and dominant isolates at treatment failure. Phylogenetic analyses revealed close genetic distances between pre-existing RAVs and dominant RAVs at treatment failure. In addition, multiple drug-resistant mutations developed on pre-existing RAVs after DCV/ASV in all non-SVR cases. In conclusion, multi-drug resistant viral clones at treatment failure certainly originated from a subpopulation of pre-existing RAVs in HCV-infected patients. Those RAVs were selected for and became dominant with the acquisition of multiple resistance-associated substitutions under DAA treatment pressure.

Hepatitis C virus (HCV) is a major cause of chronic liver disease, including liver cirrhosis and hepatocellular carcinoma^1^,^2^,^3^. The recent development of novel direct-acting antivirals (DAAs) has dramatically improved the efficacy of anti-HCV therapy and the majority of patients receiving DAA treatment achieve a sustained virological response (SVR)^4^,^5^,^6^,^7^. A subset of cases, however, fails to eradicate the viruses due to resistance-associated variants (RAVs)^8^,^9^,^10^,^11^. The first oral dual DAA therapy with the NS5A inhibitor daclatasvir (DCV) and NS3/4A inhibitor asunaprevir (ASV) was approved in Japan in 2014^12^. The 12-week SVR rate was significantly lower in patients positive for the Y93H in the NS5A region at baseline based on Sanger sequencing than in patients that were negative for the Y93H variant (43.3% vs. 90.8%, respectively). In a number of non-SVR patients, however, Sanger sequencing failed to detect the Y93H variant prior to treatment whereas the majority of viral clones were positive for Y93H at treatment failure^12^. Thus, it is assumed that low-abundant RAVs existing below the limit of detection by Sanger sequencing expanded, leading to treatment failure in patients receiving DAA therapy. Although the NS5B inhibitor sofosbuvir plus NS5A inhibitor ledipasvir combination therapy has a stronger anti-HCV effect, recent clinical studies demonstrated that treatment failure after the sofosbuvir plus ledipasvir treatment was also associated with pretreatment RAVs including NS5A-Y93H^7^. Thus, evolution of RAVs during DAA therapy is now one of the most critical challenges in clinical practice on HCV infection.

Recent progress in sequencing technology led to the development of extremely high-throughput sequencing, the so-called next-generation sequencing^13^,^14^. Using a conventional ultra-deep sequencing method with a next-generation sequencer, we previously demonstrated the detection of various low abundant viral clones and their dynamic changes in association with a variety of clinical settings in patients with hepatitis virus infection^15^,^16^. Although this technique enabled us to reveal that various RAVs naturally pre-existed in treatment-naïve hepatitis C patients^17^, it was difficult to evaluate the association between nucleotide sites mapped to different genome regions in a single viral clone, since the conventional ultra-deep sequencing is based on a multitude of shotgun methods with short reads^18^. In contrast, the recently developed third-generation sequencing (TGS) platform with single molecular real-time (SMRT) technology produces longer DNA sequences with considerably high accuracy, providing us the opportunity to obtain contiguous long sequence reads of each single viral clone^19^,^20^,^21^. In the present study, therefore, we used the TGS platform for HCV genome analysis to clarify the origin and evolution of RAVs in patients that failed to eradicate viruses by DAA treatment.

## Results

### DCV/ASV therapy for patients with chronic hepatitis or cirrhosis

A total of 316 HCV genotype 1b patients assigned to receive DCV/ASV therapy were enrolled into the study. The patients comprised 137 men and 179 women with a mean age of 68.0 (±9.2) years. Of the 316 patients, 202 had chronic hepatitis and 114 had compensated liver cirrhosis. The mean HCV-RNA level prior to the treatment was 6.1 (±1.6) logIU/mL. Before DAA therapy, Sanger sequencing detected 63 patients that were positive for RAV in NS5A, including Y93H in 55 (17.4%) and L31V/M/F/I in 11 (3.5%) cases. Among them, three had both Y93H and L31V/M/I. The remaining 253 possessed only wild-type strains. Due to the detection of RAVs at baseline, the attending physicians decided to not begin DCV/ASV therapy in 33 patients. Therefore, a total of 283 patients, including 246 without RAVs and 37 with NS5A-Y93H or L31M/V/F/I at baseline, underwent DCV/ASV therapy.

After the administration of DCV/ASV, HCV-RNA disappeared in 72.1% of patients at 4 weeks, 94.6% at 8 weeks, 95.5% at 12 weeks, and 92.9% at 24 weeks of treatment. A total of 251 (88.7%) achieved a continuous absence of detectable HCV RNA for 12 or more weeks after completion of therapy. On the other hand, 32 patients did not achieve an SVR (non-SVR); 4 non-responders, 13 with viral breakthrough, 12 with relapse after completing treatment, and 3 cases that discontinued therapy due to adverse events. The clinical features did not differ significantly between the SVR and non-SVR groups, except for previous experience with NS3 inhibitor (simeprevir)-containing therapy (0 in the SVR group vs 8 in the non-SVR group, *p* < 0.001, Table 1).

We then examined the association between the pre-treatment RAVs at Y93 and L31 of the NS5A region determined by Sanger sequencing and treatment outcome. Of 246 patients, 232 (94.3%) without any RAVs before treatment achieved an SVR, while of 37 patients positive for either Y93H and/or L31M/V/F/I at baseline, 19 (51.4%) achieved an SVR by DCV/ASV therapy. Interestingly, major viral clones at treatment failure comprised Y93H and/or L31M/V/F/I variants in all 32 non-SVR patients, including 30 cases (93.8%) with Y93H and 25 with L31M/V/F/I, whereas Sanger sequencing showed that 14 of 32 (43.8%) non-SVR patients were negative for Y93H and/or L31M/V/F/I before treatment. These findings led us to speculate that low-abundant RAVs below the limit of detection by Sanger sequencing might exist in non-SVR patients prior to DCV/ASV treatment.

### Pre-existing low-abundant RAVs detected by ultra-deep sequencing

To examine the frequency and dynamics of RAVs more sensitively, we determined the viral sequences of NS3 and NS5A regions using conventional ultra-deep sequencing. First, we conducted a control experiment to validate the efficacy and error rates in ultra-deep sequencing of the viral genome. For this purpose, we used an HCV-containing plasmid as a template and amplified its NS5A region, as described in the Materials and Methods. The ultra-deep sequencing platform provided us information about the 420 base pairs (bp) of the NS5A genome derived from plasmids with a mean coverage depth of 57,831 reads at each nucleotide site. The overall mismatch error rate by multiplex ultra-deep sequencing was a mean of 0.0012 (SD = 0.0010, 95%CI: 0.0004–0.0031) per bp (Supplementary Figure S1). Taking the value of the mean + 2SD mismatch error rate into consideration, we determined the threshold to detect a true RAV as 0.0040 per bp.

**Figure 1:**
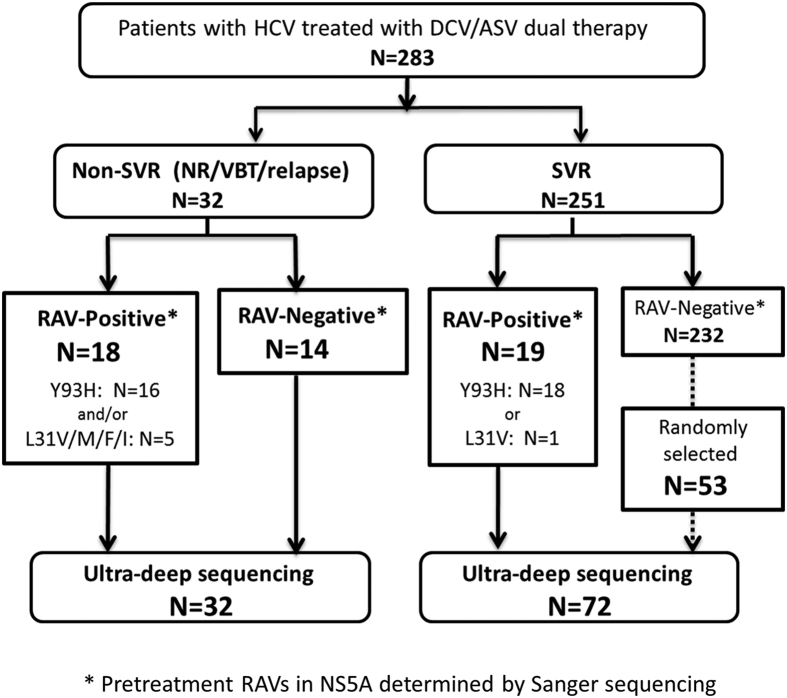
Flow chart showing the treatment outcome and patient selection. The treatment effect of HCV-infected patients receiving DCV/ASV and the patient selection process for ultra-deep sequencing analyses are represented in the flow chart. *Pre-treatment RAVs in NS5A region determined by Sanger sequencing. N, number of patients. DCV, daclatasvir; ASV, asunaprevir.

Serum samples from 104 patients were applied to ultra-deep sequencing, including all 32 non-SVR patients, 19 SVR patients with pre-existing RAVs at Y93 and/or L31 in NS5A, and 53 randomly selected SVR patients without pre-existing NS5A-RAVs, determined by Sanger sequencing (Fig. 1). Ultra-deep sequencing identified Y93H in 66 (63.5%) and L31M/V/F/I in 23 (22.1%) of the 104 pretreatment samples, while Y93H and L31M/V/F/I were detected by Sanger sequencing in 34 (32.7%) and 6 (5.8%) cases, respectively. Among the patients in whom NS5A-RAVs were not detected by pretreatment Sanger sequencing, Y93H-positive clones were detected by ultra-deep sequencing in 10 of 14 (71.4%) non-SVR patients, compared with 20 of 53 (37.7%) SVR patients (*p* = 0.035, Fisher’s exact test) (Supplementary Figure S2). These findings suggested that ultra-deep sequencing achieved sensitive detection of resistance-associate substitutions of the viral genome and could detect low-abundant RAVs that were present at baseline, especially in non-SVR patients receiving DCV/ASV.

### Dynamics of RAVs in non-SVR patients receiving DCV+ ASV dual therapy

We then evaluated the viral dynamics of RAVs in non-SVR patients in detail. Although Sanger sequencing was positive for Y93H in only 16 of 32 (50.0%) patients before treatment, ultra-deep sequencing detected the Y93H variant in 28 (87.5%) of 32 non-SVR patients (Supplementary Table S1). The remaining four cases in whom Y93H was negative at baseline by ultra-deep sequencing possessed other RAVs, including NS3-Q80R/K in two and NS3-D168V/E/A/F in two patients. At treatment failure, 29 of 32 (90.6%) non-SVR cases, including 12 cases in whom Sanger sequencing was negative for Y93H at baseline, possessed Y93H as a major viral clone with a mean frequency of 70.6% (21.9–99.8%) among total circulating viruses. Moreover, RAVs at NS5A-L31 and/or NS3-D168 emerged in the majority of non-SVR patients in whom ultra-deep sequencing had not detected these RAVs at baseline. Indeed, NS5A-L31V/M/F/I was detected in 27 (84.4%) and NS3-D168V/E/A/F in 29 (90.6%) of 32 non-SVR patients (Supplementary Table S1).

**Figure 2:**
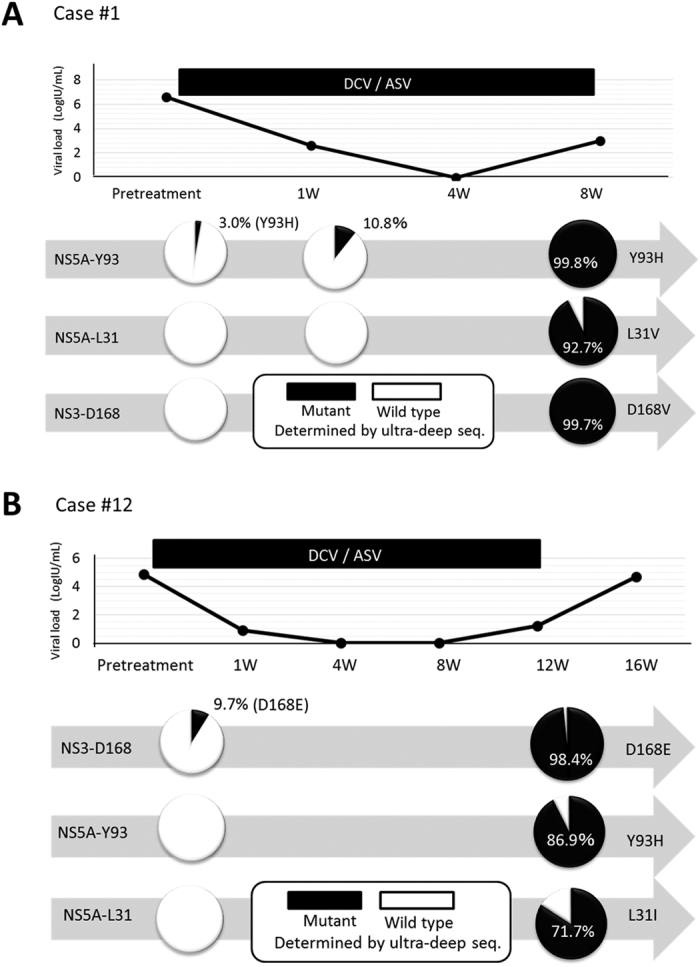
Clinical courses of two representative non-SVR cases in whom pretreatment RAVs were not detected by Sanger sequencing. Time courses of HCV-RNA levels are shown in the upper panels. Every pie chart in the lower panels demonstrates the amino acid frequencies at NS3-D168, NS5A-L31, and Y93 determined by ultra-deep sequencing. (**A**) A 61-year-old female with hepatitis C (Case #1). HCV-RNA became undetectable 4 weeks after the initiation of therapy and increased again at week 8 of daclatasvir plus asunaprevir (DCV/ASV) therapy. (**B**) A 72-year-old female with liver cirrhosis (Case #12). She experienced a viral breakthrough 11 weeks after the initiation of DCV/ASV therapy.

Representative clinical courses of non-SVR cases are shown in Fig. 2. In Case#1, after HCV-RNA became undetectable 4 weeks after the initiation of therapy, HCV-RNA levels increased again at week 8 of therapy. At viral breakthrough, ultra-deep sequencing demonstrated the expansion of HCV clones, with Y93H reaching 99.8% of the circulating viral isolates. Although Sanger sequencing was negative for Y93H at baseline, ultra-deep sequencing analysis revealed a pretreatment serum Y93H frequency of 3.0% that was concentrated to 10.8% at day 7 of treatment (Fig. 2A). In contrast to Case#1, ultra-deep sequencing did not detect Y93H-positive clones at baseline in Case#12, she failed to achieve SVR because of the appearance of Y93H-positive RAVs after DAA therapy. Of note, low-abundant (9.2%) NS3-D168E clones were present at baseline, and the patient experienced viral breakthrough with the expansion of D168E-positive clones 11 weeks after beginning DCV/ASV therapy (Fig. 2B). These findings suggested that a certain type of low-abundant drug-resistant viral clones at baseline might selectively expand after administering DCV/ASV in each non-SVR patient.

On the other hand, although RAVs at NS5A-L31 and NS3-D168 were undetectable by ultra-deep sequencing prior to therapy in Case#1, these RAVs emerged as the major viral population at relapse (Fig. 2A and Supplementary Table S1). A similar finding was observed in Case#12. Almost all HCV clones possessed NS5A-Y93H at viral breakthrough, while the Y93H clone had not been detected by ultra-deep sequencing prior to treatment (Fig. 2B and Supplementary Table S1). These findings suggested that multiple drug-resistant mutations might newly emerge on pre-existing viral clones after the administration of DAAs.

### The TGS platform determined population diversity with a high degree of genetic heterogeneity in HCV clones

**Figure 3:**
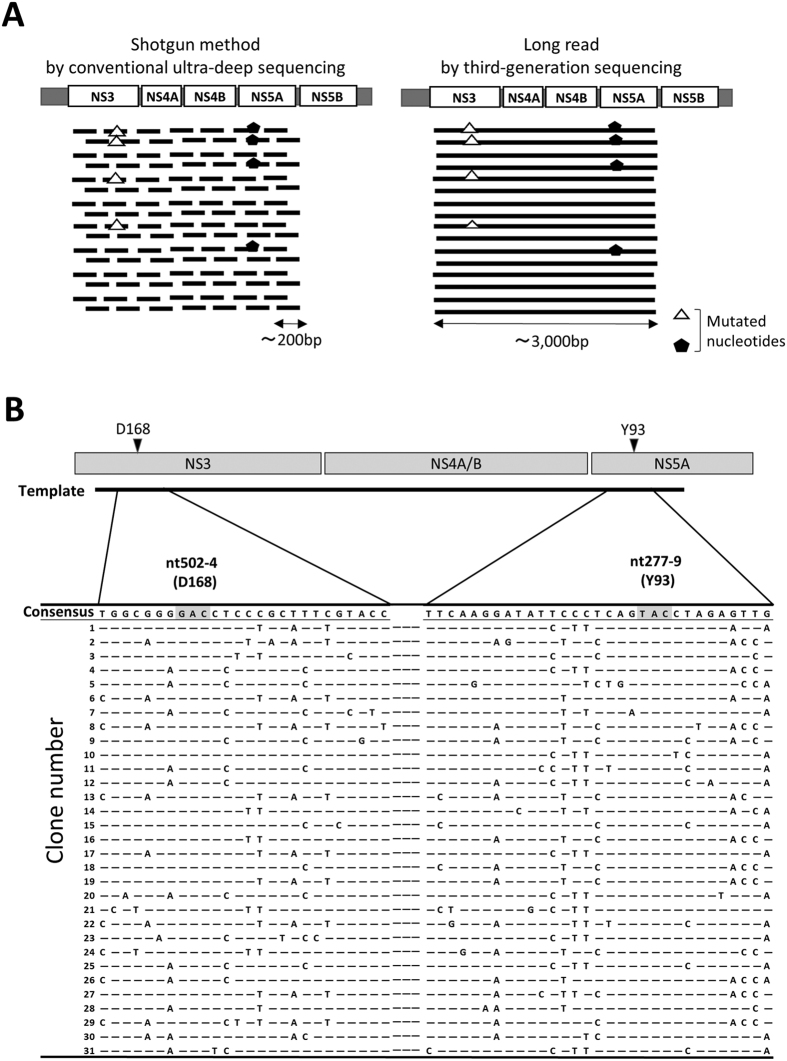
Viral genome sequences in the NS region determined by third-generation sequencing (TGS) platform. (**A**) Schematic diagrams of the sequencing reads determined by the shotgun method using conventional ultra-deep sequencing (left panel) and contiguous long reads obtained by TGS (right panel). (**B**) Genetic diversity of the genotype 1b HCV in chronically infected patients before treatment. Distribution of nucleotide alterations around nucleotide (nt)#502 (corresponding to amino acid D168) in the NS3A and nt#277 (corresponding to amino acid Y93) in NS5A regions in Case#3.

To clarify whether RAVs occupying almost all HCV strains on relapse emerged from a subpopulation of low-abundant drug-resistant clones prior to therapy or were newly acquired during therapy, it is essential to obtain contiguous long sequence reads of each viral isolate to evaluate the co-occurrence of nucleotide variations. Therefore, we applied the TGS technique to determine the contiguous viral sequences spanning the NS3 to NS5A region (Fig. 3A).

First, we performed control sequencing using an HCV-containing plasmid as a template and amplified its NS3/4 and NS5A regions. The SMRT sequencing platform provided a total of 127,200 raw reads (corresponding to 853 Mb data size) covering 3946 bp of the NS3/4 and 5 A genome regions derived from the plasmids. Among the total Circular Consensus Sequence (CCS) reads generated with the custom PacBio RSII program^21^,^23^, only CCS reads making 10 or greater passes around the closed loop SMRTbells (10-pass CCS reads) were strictly selected to obtain a high accuracy of sequence reads (Supplementary Figure S3). The 10-pass CCS reads were generated from the sequencing of each identical template at least 10 times, and the final consensus sequence of the target template was determined according to the default workflow (Supplementary Figure S4). As a result, the overall mismatch error rate of 10-pass CCS reads was a mean of 0.000287 (SD = 0.000771, 95%CI: 0.000226–0.000270) per bp, indicating an extremely high accuracy of sequence reads achieved by the current SMRT sequencing platform (Supplementary Table S2).

**Figure 4:**
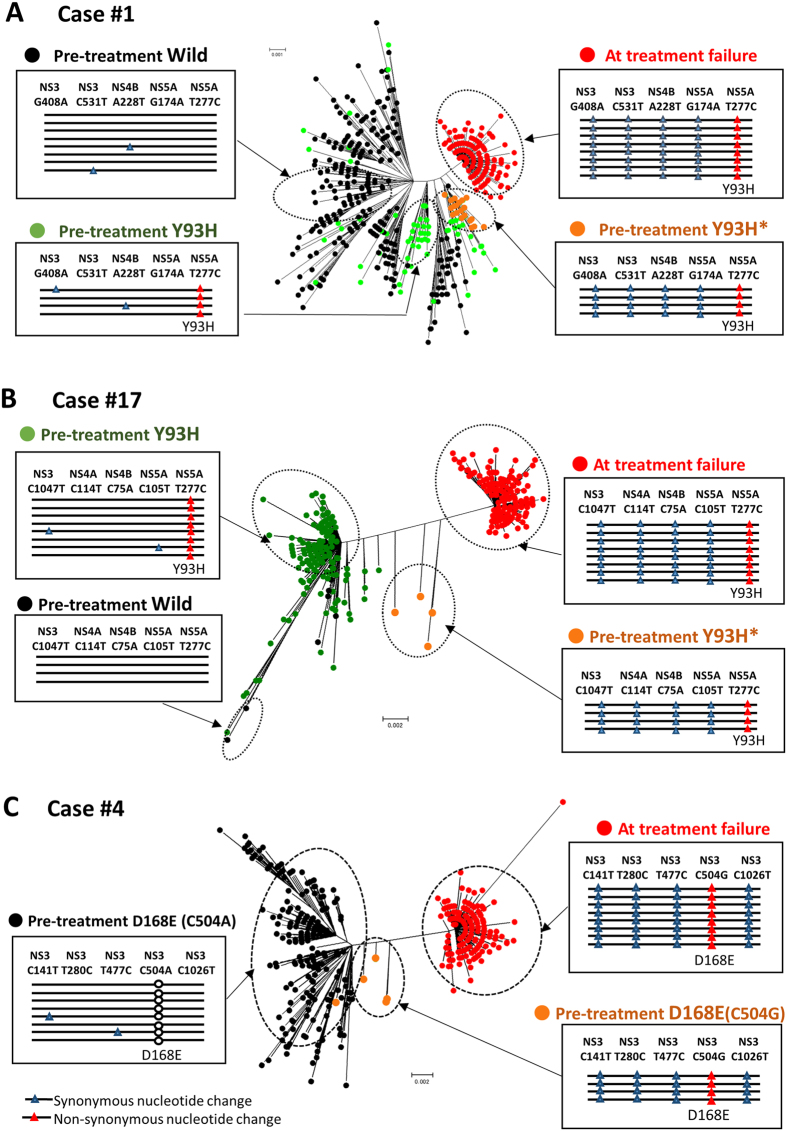
Phylogenetic and linkage analyses of the pretreatment and posttreatment HCV clones in non-SVR cases. Phylogenetic trees showing the pretreatment and posttreatment HCV clones with and without specific drug-resistant mutations (**A**. Case#1. **B**. Case#17. **C**. Case#4) Linkage of four synonymous nucleotide alterations (blue triangle) with T277C corresponding to amino acid Y93H (red triangle) (**A** and **B**), and C504G corresponding to amino acid D168E (red triangles) (**C**) are detectable in a subset of viral isolates at baseline and the majority of RAVs at treatment failure. In phylogenetic trees, orange dots show a subset of the pretreatment Y93H-positive clones with the synonymous nucleotide alterations that were detectable in the majority of RAVs at treatment failure (**A** and **B**). In Case#4, orange dots indicate a subset of the pretreatment D168E(C504G)-positive clones with the four synonymous nucleotide alterations that were detectable in the majority of RAVs at treatment failure (**C**).

**Figure 5:**
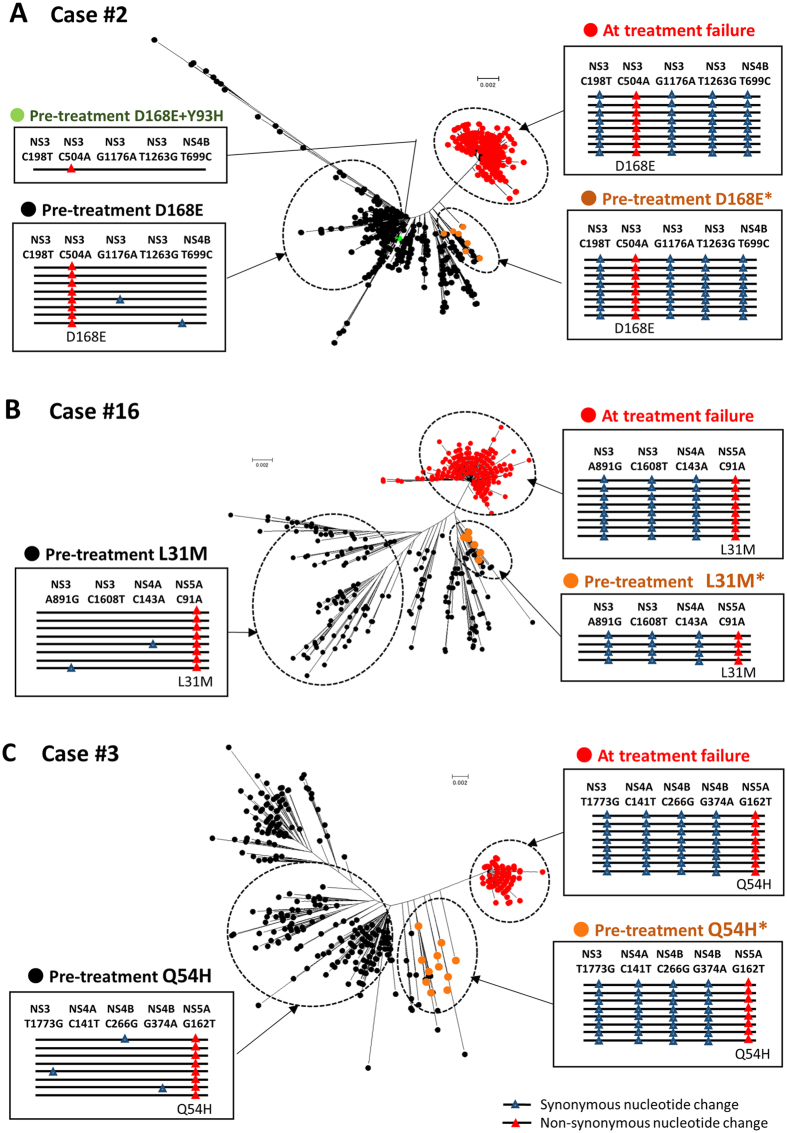
Phylogenetic and linkage analyses of the pretreatment and posttreatment HCV clones in non-SVR cases. Phylogenetic trees showing the pretreatment and posttreatment HCV clones with and without specific drug-resistant mutations (**A**. Case#2. **B**. Case#16. **C**. Case#3). Several synonymous nucleotide alterations (blue triangles) co-existed with C504A corresponding to amino acid D168E (red triangles) (**A**), C91A corresponding to L31M (red triangles) (**B**), and G162T corresponding to Q54H (red triangles) (**C**) in a subset of pretreatment viral isolates and the majority of RAVs at treatment failure. In phylogenetic trees, orange dots indicate the pretreatment RAVs with the synonymous nucleotide alterations that were detectable in the majority of RAVs at treatment failure. Black dots indicate the pretreatment RAVs without these synonymous nucleotides.

We then applied SMRT sequencing on 12 paired samples from 6 non-SVR cases (Table 2). The reference sequences of the population average clone in each serum specimen were constructed using the Sanger sequencing method. When HCV sequence reads were aligned to each reference sequence, the coverage curve of every nucleotide position showed a uniform distribution compared with the coverage curve in the IonProton platform, indicating that the SMRT sequencing platform provided contiguous viral sequences between the NS3, 4A/B, and NS5A regions for each specimen. Compared with each reference sequence of the population average clone, the viral mutations in each viral isolate were almost evenly distributed throughout the NS3, 4A/B, and 5A regions (Fig. 3B and Supplementary Figure S5). The landscape of the HCV population was investigated by constructing phylogenetic trees from the viral isolate sequences with 10-pass CCS reads derived from six serum samples at baseline (mean of 285 clones; range, 196–508). Phylogenetic tree analysis revealed that all viral isolates present at baseline were separately and widely distributed, accompanied by the formation of small clusters, and every viral clone identified in each sample showed sequence diversity (Figs 4 and 5, and Supplementary Table S3). Of note, none of the viral clones shared completely identical sequences of the NS3, 4A/B, and 5A regions in the viral population of each serum (Fig. 3B). These findings suggested that viral quasispecies in HCV patients comprised a substantially high population diversity, in which none of the viral isolates identified shared identical viral genome sequences in the NS3, NS4A/B, and 5A regions.

### Evolution of RAVs determined by TGS analysis in patients receiving DCV/ASV treatment

To gain insight into the origin of RAVs at treatment failure, we focused on known clinically relevant resistance-associated substitutions in the NS3 (V36, T54, Q80, R155, A156, and D168) and the NS5A (L28, R30, L31, P32, Q54, P58 and Y93) regions^10^,^24^, and compared the sequence reads obtained by TGS analyses at baseline and at treatment failure in association with these RAVs in six non-SVR cases. For this purpose, we divided the FASTQ data of each sample into each RAV-positive and -negative long reads, followed by phylogenetic analysis using all 10-pass CCS reads of pretreatment and posttreatment viral clones. Because the sequence reads provided the linkage information between resistance-associated substitutions and every individual polymorphism on the same viral isolate, we also determined the synonymous nucleotide alterations co-existing with each resistance-associated substitution to identify the origin of RAVs detected at treatment failure based on the linkage information of resistance-associated substitutions and other synonymous polymorphisms.

First, we performed linkage analysis and observed the co-occurrence of several synonymous mutations with a certain resistance-associated substitution in a sub-population of RAVs present at baseline and dominant isolates at treatment failure in all non-SVR cases examined. In Case#1, 96.3% (208/216) of the viral clones at treatment failure possessed Y93H (nucleotide substitution of NS5A-T277C) and four additional synonymous nucleotide changes, NS3-G408A, NS3-C531T, NS4B-A228T, and NS5A-G174A (Fig. 4A). These four nucleotide polymorphisms were concurrently present in 16.1% (15/93) of the pre-treatment Y93H-positive viral isolates, while none of the Y93H-negative HCV clones were simultaneously present in these four nucleotide variations (p < 0.0001). Similarly, Case#17 had the concurrent presence of 12 nucleotide variations, such as NS3-C1047T, NS3-T1188C, NS4A-C114T, NS4B-C75A, and NS5A-C105T in a subpopulation of Y93H-positive isolates at baseline and a majority of Y93H-positive isolates at relapse, but not in Y93-wild clones at baseline (Fig. 4B). Concurrent linkage of a D168E-associated mutation and several synonymous nucleotide variations were commonly observed in pre-existing clones and those at treatment failure in two cases (Cases #2, #4). Interestingly, Case#4 possessed D168E-positive RAVs derived from the nucleotide substitution of either C504A or C504G at baseline. At treatment failure, all the viral isolates had C504G simultaneously with several synonymous nucleotide variations that were commonly detected in low-abundant C504G-positive clones at baseline (Fig. 4C). Consistently, in these cases phylogenetic analysis revealed a close genetic distance between the pretreatment and posttreatment RAVs that carried a common set of synonymous nucleotide variations (Figs 4C and 5A). The remaining two cases (Cases #3, #16) had similar findings, indicating that viral clones at treatment failure shared the same synonymous mutations linked with resistance-associated substitutions, L31M/I and/or Q54H, with those detected at baseline (Figs 5B and C).

**Figure 6:**
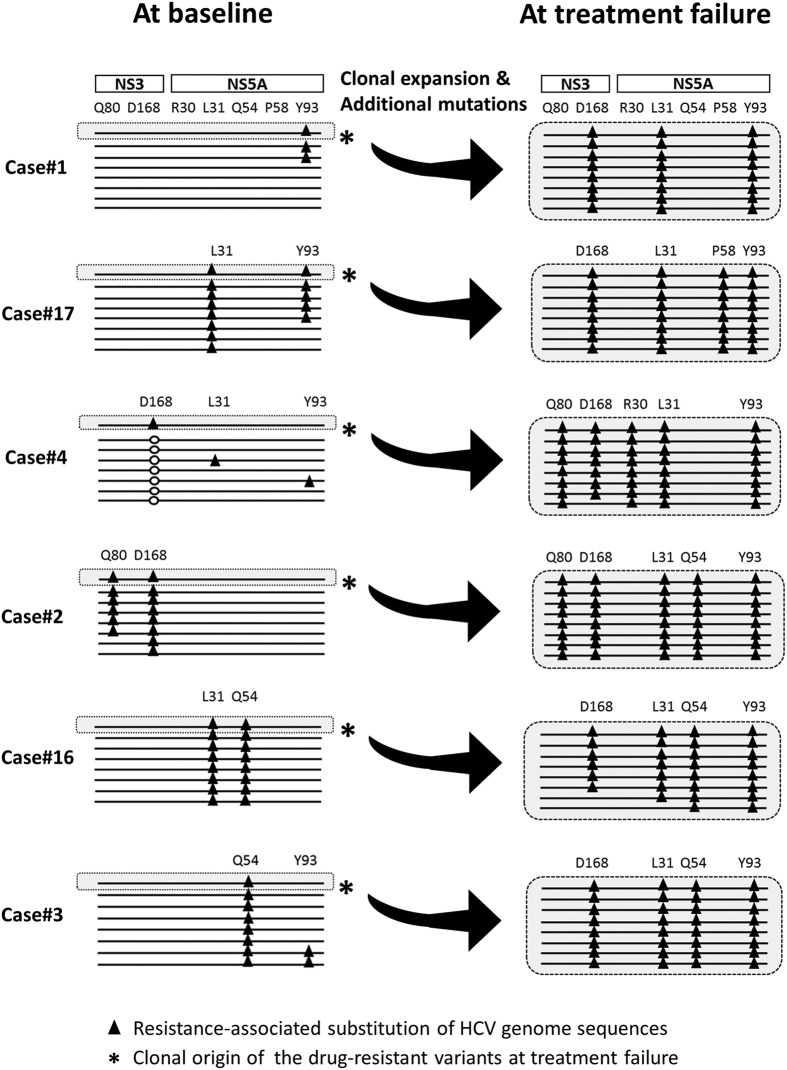
Schemas representing the evolution of RAVs focusing on known resistance-associated substitutions in the NS3 and NS5A regions. The distribution patterns of seven resistance-associated alterations in amino acids (NS3-Q80, D168, NS5A-R30, L31, Q54, P58, and Y93) determined by third-generation sequencing platform are demonstrated. Schematic diagrams of pretreatment samples are shown in the left panels and those at treatment failure in the right panels. *White circle in Case#4 show the D168E caused by C504A nucleotide substitution, which was not detectable in the viral isolates at treatment failure.

Of note, RAVs that were already present at baseline also acquired multiple resistance-associated substitutions during DAA treatment in all non-SVR cases (Fig. 6). For example, Case#1 showed that the majority of clones with Y93H and the co-existing four synonymous nucleotide changes additionally possessed D168V in NS3 and L31V in NS5A regions at treatment failure, indicating that low-abundant Y93H-positive clones at baseline newly acquired these resistance-associated substitutions during DCV/ASV treatment (Fig. 6). These findings suggested that RAVs at treatment failure were selected for and became dominant with newly acquired multiple resistant mutations under DAA treatment pressure.

## Discussion

Viral clones containing resistance-associated substitutions are currently one of the most serious problems for anti-viral therapies in HCV-infected patients^10^,^11^. In this study, we analyzed the dynamics of RAVs in patients receiving DAA treatment, especially focusing on patients who failed to eradicate viruses by oral DCV/ASV therapy. We demonstrated that ultra-deep sequencing analyses achieved sensitive and quantitative detection of drug-resistant HCV isolates in patients during DCV/ASV therapy. The newly developed TGS technique has revolutionized genomic analyses, allowing for highly accurate and long contiguous genome sequencing^19^,^23^,^25^. Therefore, we optimized the TGS platform based on the SMRT sequencing approach to HCV genome analysis and determined contiguous viral sequences spanning the NS3/4 to NS5A regions at baseline and at treatment failure in non-SVR patients by DCV/ASV therapy.

Among the patients receiving DCV/ASV therapy, 32 of 283 (11.3%) cases failed to eradicate HCV accompanied by the emergence of RAVs such as Y93H- and/or L31M/V/F/I-positive isolates as a major population, suggesting that those RAVs were associated with treatment failure against DCV/ASV. Although population sequencing by the Sanger method detected RAVs in only a subset of non-SVR patients before treatment, ultra-deep sequencing analyses efficiently detected low-abundant RAVs that already existed at baseline in non-SVR cases in whom pretreatment Sanger sequencing did not detect those isolates. A Y93H–positive clone was undetectable in the remaining 4 of 32 (12.5%) non-SVR patients before DAAs administration, but those cases possessed resistance-associated substitutions in the NS3 region, including D168V/E/A/F and Q80R in the low-abundant viral clones. These findings suggest that RAVs occupying the serum of non-SVR patients at treatment failure might emerge from drug-resistant clones that existed prior to the therapy.

To determine the clonal origin as well as the dynamics of RAVs related to treatment failure, we used, for the first time, the TGS platform for HCV genome sequencing. SMRT technology is the first commercially available platform to directly observe single molecules of DNA polymerase as they synthesize DNA sequences, achieving kilobase-long reads and overcoming the limitation of conventional NGS with short reads. In the present study, we determined the read length of the target amplicon to be approximately 3000 bp spanning the NS3/4 and NS5A regions, and obtained extremely high accuracy (>99.97%) of each sequencing read by strictly selecting the 10-pass CCS reads that were generated by the sequencing of each identical template more than 10 times^19^. Using this TGS platform, we determined the contiguous viral sequences spanning the NS regions and revealed that viral quasispecies in each patient exhibited substantially high population diversity with a variety of nucleotide changes distributed all over the viral genomic regions. Of note, none of the viral clones identified in each case shared completely identical sequences of the whole NS regions, suggesting that HCV infection is characterized by an extremely versatile virus population consisting of viral clones that differ from one another with various widely distributed nucleotide substitutions. Although the conventional ultra-deep sequencing method also revealed the genetic heterogeneity of HCV^17^,^26^,^27^, the multitudinous short reads made it difficult to evaluate the linkage between nucleotide sites located in different viral genome regions. The TGS platform enabled us to determine the contiguous genome sequences of each single viral clone and provided evidence for the massive genetic heterogeneity of the infecting viruses.

Based on the highly accurate sequences obtained by the TGS platform, we examined the linkage of resistance-associated substitutions with co-existing synonymous mutations in each viral isolate and investigated the origin of the RAVs observed at treatment failure. Linkage analyses demonstrated the co-occurrence of several synonymous mutations with a certain resistance-associated substitution in RAVs at baseline and dominant isolates at treatment failure, suggesting that low-abundant viral clones with certain resistance-associated substitutions at baseline expanded during DAA treatment in each case. Indeed, RAVs in the serum at treatment failure derived from pre-existing Y93H-positive clones in two cases (Cases #1, #17), D168E-positive isolates in two cases (Cases #2, #4), and L31M/I and/or Q54H-positive clones in two cases (Cases #3, #16). The phylogenetic analysis supported these findings, revealing that a subpopulation of RAVs present at baseline were closely genetically related with the RAVs detected as the major population at treatment failure in each non-SVR case. Our current findings are consistent with the recently published study using SMRT sequencing on HIV patients with treatment failure, showing that drug-resistant viral isolates already existed as a minor species in the HIV population before treatment^28^. On the other hand, we must also draw attention to the fact that multiple drug-resistant mutations undetectable by deep sequencing at baseline also newly emerged at treatment failure. For example, RAVs at treatment failure that originated from pre-existing Y93H-positive clones possessed not only Y93H but also NS5A-L31V and NS3-D168V, while the pre-existing Y93H-positive clones did not possess NS5A-L31V and NS3-D168V in the serum of Cases #1 and #17. Similar findings were observed in the remaining non-SVR cases, suggesting that multiple resistance-associated substitutions are likely to newly develop on the pre-existing viral clones during DAA treatment.

Consistent with our findings, recent studies revealed that RAVs with multi-drug resistance within the NS3/NS4A and NS5A domains emerge in non-SVR patients receiving DAA-contained regimens^11^,^29^,^30^. What has to be noticed is that those RAVs with multi-drug resistance could cause the treatment failure to newly developed DAAs. For example, multiple drug-resistant mutations were detected in the pretreatment serum of those failing to sofosbuvir plus ledipasvir^22^,^31^. In addition, SVR12 rate was significantly decreased in patients receiving sofosbuvir plus ledipasvir after failing to daclatasvir plus asunaprevir, when they had NS5A multiple drug-resistant mutations at retreatment baseline^32^. Thus, it cannot be emphasized too strongly that developing a detection method of multi-drug resistance in viral clones would be urgently required to achieve the eradication of HCV even in patients receiving the retreatment after a DAA-containing treatment failure. In this regard, the advantage of contiguous long sequence reads obtained by TGS methodology would be a powerful tool for the detection of multiple drug-resistant mutations in each single viral clone.

One of the limitations of the current TGS platform is the limited number of viral clones identified because of the strict selection of the identified sequence reads with very high accuracy. Therefore, further optimization of the TGS sequencing analysis might be necessary to fully unveil the landscape of viral heterogeneity of the infecting HCV.

In conclusion, ultra-deep sequencing was useful for detecting low-abundant pre-existing RAVs in non-SVR patients in whom pretreatment Sanger sequencing could not detect drug-resistant substitutions at baseline. TGS sequencing analyses based on the SMRT platform demonstrated that a subpopulation of pre-existing RAVs can survive and become dominant clones at treatment failure in non-SVR patients receiving DAA. In addition, multiple drug-resistant substitutions newly emerge under treatment pressure by DAAs, selectively expand with survival benefit, and acquire additional RAVs, thereby producing multi-drug resistant clones as the major HCV clones in the serum of patients at treatment failure. The importance of detecting low-abundant RAVs prior to DAA therapy cannot be overemphasized because post-treatment RAVs could originate from the pre-existing RAVs in HCV-infected patients receiving DAA treatment. Simultaneously, we should also recognize that drug-resistant mutations sometimes newly emerge during DAA therapy, suggesting that it is also difficult to fully predict treatment outcome by detecting RAVs in HCV-infected patients receiving DAA therapy.

## Methods

### Patients and samples

Patients with genotype 1b HCV infection who were assigned to receive DCV/ASV therapy at Kyoto University and 11 cooperative institutions were enrolled in this study from September through December 2014. DCV/ASV therapy was carried out with 60 mg DCV once a day and 200 mg ASV twice a day for 24 weeks. Serum samples were collected from all patients on enrollment and at treatment failure. HCV-RNA was measured using Taqman reverse transcription-polymerase chain reaction (RT-PCR; ThermoFisher Scientific, Waltham, MA, USA). Representative RAVs (NS5A-Y93H and L31V/M/F/I) were examined by population-based Sanger sequencing, as described previously^15^. Detection of HCV variants in Sanger sequencing were determined by visual inspection of the electrophrogram using Sequencher software (Gene Codes Corporation, Ann Arbor, MI, USA). Written informed consent was obtained from each patient prior to DCV/ASV therapy. All protocols were approved by the ethics committee of Kyoto University. This study protocol complied with all provisions of the Declaration of Helsinki.

### Conventional ultra-deep sequencing

Total-RNA was extracted from serum using the QIAquick Viral Mini kit (Qiagen, Valencia, CA, USA). Reverse transcriptase (RT) reaction was performed using the One Step RT-PCR kit (Takara Bio, Shiga, Japan) and the NS5A region of the HCV genome sequences was amplified using Phusion High-Fidelity DNA polymerase (ThermoFisher Scientific) and the primers shown in Supplementary Table S4. QIAquick gel extraction kit and QIAquick PCR purification kit (Qiagen) were used for the purification of PCR products.

Ultra-deep sequencing with multiplexed tags was performed using the Ion Proton Sequencer (Thermo Fisher Scientific) according to the manufacturer’s protocol^15^. In brief, libraries were generated using 100 ng of genomic DNA and an Ion Xpress Plus Fragment Library Kit comprising the Ion Shear Plus Reagents Kit. Amplicons were ligated to adapters from the Ion Xpress Barcode Adapters. The size range was visualized using TapeStation with a DNA 1000 kit (Agilent, Santa Clara, CA, USA), and an Ion Library Taqman Quantitation Kit was used to determine the library concentration for the preparation of the emulsion PCR. Each library was diluted and then used as a template for clonal amplification on Ion Sphere particles during emulsion PCR according to the Ion PI Template OT2 200 Kit User Guide. Sequencing was conducted over 400 sequencing cycles with the Ion PI Sequencing 200 Kit v3 on an Ion PI Chip (Life Technologies). The above-mentioned protocol resulted in an average read length of 150 nucleotides.

Sequence data analyses were conducted using high-performance alignment software NextGENe (ver. 2.4.3) (SoftGenetics, State College, PA, USA) as described previously^14^,^15^. The sequence reads obtained from the IonProton Sequencer were aligned with reference sequences for the entire HCV genome (GenBank; D90106.1). Reads with ≥85% of bases matching a particular position in the reference sequences were aligned. Each position of the viral genome was assigned a coverage depth, representing the number of times the nucleotide position was sequenced.

### Single molecular real-time (SMRT) sequencing (Third-generation sequencing)

SMRT sequencing was conducted using a PacBio RSII sequencer according to the manufacturer’s protocol (Pacific BioScience, Menlo Park, CA, USA)^19^,^20^. In brief, 3120-bp viral sequences spanning NS3 and NS5A regions of HCV genome were amplified using the PrimeScript One Step RT-PCR kit (Takara) and PrimeSTAR HS kit (Takara). Purified DNA product (5 μg) was used to construct the PacBio DNA library using the PacBio standard template prep protocol. The samples were sequenced on the PacBio RSII platform on a single SMRT Cell per sample. P6C4 polymerase was used for the sequencing reaction and 6-h movie windows were used for signal detection. After raw sequence data were generated, base-calling and Circular Consensus Sequence (CCS) read generation were performed using version 2.3.0.1 of PacBio’s instrument control and SMRT Analysis software with default parameters. To obtain high accuracy of the sequence reads, we selected CCS reads making at least 10 passes around the closed loop SMRTbells (10-pass CCS reads). The output sequences are in FASTQ format.

To evaluate the linkage of each nucleotide change on the identical sequence reads and to generate phylogenetic trees, long CCS reads (>3000 bp, in one case >2000 bp) were selected using R ver. 2.2.1 software. Selected 10-pass CCS reads in FASTA file format were aligned with Clustal W software, and then diversity analysis and phylogenetic and linkage analysis were conducted. The evolutionary history was inferred using the Maximum Likelihood method. Evolutionary analyses were conducted in MEGA7 and Microsoft Excel^33^.

### Statistical analysis

Categorical variables were analyzed by Fisher’s exact test. Continuous variables were analyzed using the Mann-Whitney U test. Data were analyzed using R ver.2.2.1. Two-tailed probability values of *p* < 0.05 were considered significant.

## Additional Information

**Accession codes:** Sequence reads were deposited in the DNA Data Bank of Japan Sequence Read Archive (http://trace.ddbj.nig.ac.jp/dra/index_e.shtml) under accession number DRA005147.

**How to cite this article:** Takeda, H. *et al*. Evolution of multi-drug resistant HCV clones from pre-existing resistant-associated variants during direct-acting antiviral therapy determined by third-generation sequencing. *Sci. Rep.* **7**, 45605; doi: 10.1038/srep45605 (2017).

**Publisher's note:** Springer Nature remains neutral with regard to jurisdictional claims in published maps and institutional affiliations.

## Supplementary Material

Supplementary Materials

## Figures and Tables

**Table 1:** Baseline characteristics of non-SVR and SVR patients receiving DCV/ASV therapy.

Variable		Non-SVR (n = 32)	SVR (control) (n = 72)	*p*
Age (years)	Median (Range)	67.5 (43–81)	69.5 (42–82)	0.896^a^
Sex	Male/Female	12/20	27/45	0.386^b^
Liver cirrhosis	Yes/No	15/17	24/48	0.197^a^
HCV-RNA (logIU/mL)	Median (Range)	6.35 (3.6–7.3)	6.40 (5.2–7.9)	0.523^b^
Pretreatment ALT level (U/L)	Median (Range)	38 (13–123)	43 (6–183)	0.717^a^
Pretreatment T-BIL level (mg/dL)	Median (Range)	0.7 (0.4–2.9)	0.8 (0.4–8.2)	0.119^a^
Pretreatment Platelet (*10^4^/L)	Median (Range)	14.1 (4.1–44.6)	12.7 (4.4–34.1)	0.612^a^
Previous anti-HCV therapy				
IFN monotherapy	Yes/No	5/27	3/69	0.103^b^
PEG-IFN plus RIB	Yes/No	22/10	46/26	0.663^b^
TRV plus PEG-IFN RIB	Yes/No	1/31	5/67	0.664^b^
SMV plus PEG-IFN RIB	Yes/No	8/24	0/72	<0.0001^b^
Pretreatment RAVs (Sanger sequencing)				
NS5A-Y93H	+/−	1/16	18/54	0.022^b^
NS5A-L31M/V/I	+/−	5 /27	1/71	0.010^b^

ALT, alanine aminotransferase; T-BIL, total-bilirubin; IFN, interferon; PEG-IFN, pegylated interferon; RIB, ribavirin; TRV, telaprevir; SMV, simeprevir; RAV; resistance-associated variant.

Data are the number or median (range); ^a^Mann Whitney U test, ^b^Fisher’s exact test.

**Table 2:** Selection of highly accurate sequence reads (10-pass CCS reads) in 12 paired samples from 6 non-SVR cases.

Case #		Data size of total raw reads (FASTQ, Mb)	Data size of total 10-pass CCS read (FASTQ, Mb)	Base number of total 10-pass CCS read	Base number of selected 10-pass CCS reads*	# of selected 10-pass CCS reads
#1	Pre	1,803	7.7	3,884,777	1,176,880	375
	Post	197	3.8	1,643,899	682,340	218
#2	Pre	1,259	17.9	8,989,951	1,539,240	508
	Post	1,696	25.3	12,746,272	1,639,230	541
#3	Pre	955	11.1	5,518,321	598,500	285
	Post	1,245	10.8	5,440,045	615,300	293
#4	Pre	372	3.8	1,864,548	605,775	197
	Post	2,177	30.8	15,594,271	759,525	247
#16	Pre	2,194	41.0	21,887,194	611,716	196
	Post	2,387	16.6	8,355,954	955,026	306
#17	Pre	2,613	35.5	17,878,934	657,090	210
	Post	1,118	20.2	10.158,188	704,025	225

CCS, circular consensus sequence; SMRT, single molecule real time; pre, pretreatment; post, at treatment failure; *, >3000 bp-long reads aligned to genotype 1b HCV genome sequence (>2000 bp-long reads in Case#3).
