# Supplementary material for: Evolution of multi-drug resistant HCV clones from pre-existing resistant-associated variants during direct-acting antiviral therapy determined by third-generation sequencing
Source: Sci Rep. 2017 Mar 31;7:45605. doi: 10.1038/srep45605 (PMC5374541; doi:10.1038/srep45605)
Supplement: Supplementary Materials [file srep45605-s1.pdf]

Supplementary Information

Evolution of multi-drug resistant HCV clones from pre-existing resistant-associated variants during direct-acting antiviral therapy determined by third-generation sequencing

Haruhiko Takeda[1], Yoshihide Ueda[1], Tadashi Inuzuka[1], Yukitaka Yamashita[2], Yukio Osaki[3], Akihiro Nasu[3], Makoto Umeda[4], Ryo Takemura[5], Hiroshi Seno[1], Akihiro Sekine[6,7] and Hiroyuki Marusawa[1]

[1]Department of Gastroenterology and Hepatology, Graduate School of Medicine, Kyoto University, Kyoto, Japan

[2]Department of Gastroenterology and Hepatology, Japanese Red Cross Wakayama Medical Center, Wakayama, Japan

[3]Department of Gastroenterology and Hepatology, Osaka Red Cross Hospital, Osaka, Japan

[4]Department of Gastroenterology and Hepatology, Hyogo Prefectural Amagasaki General Medical Center, Hyogo, Japan

[5]Clinical Research Center, Chiba University Hospital, Chiba, Japan

[6]Center of Preventive Medical Sciences, Chiba University, Chiba, Japan

[7]Department of Genomic Medicine Omics Research Center, National Cerebral and Cardiovascular Center, Osaka, Japan

Supplementary Figure S1

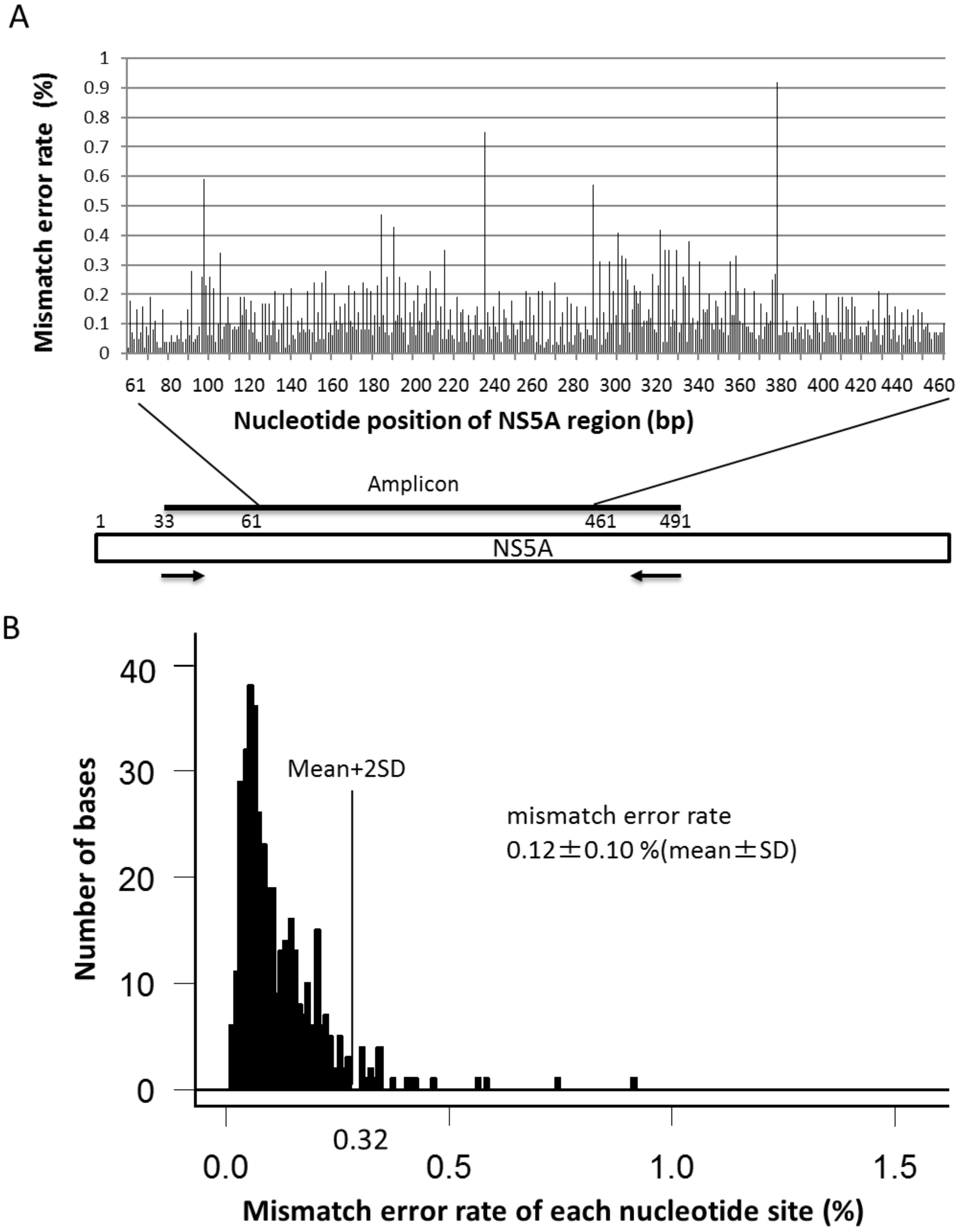

Supplementary Figure S1. Mismatch error frequency of ultra-deep sequencing for the plasmid encoding the HCV sequence. (**A)** Mismatch error rate of each nucleotide position in the NS5A region of the HCV-containing plasmid determined by ultra-deep sequencing. (**B)** Histogram of the mismatch error rate based on the data shown in panel **A**.

Supplementary Figure S2

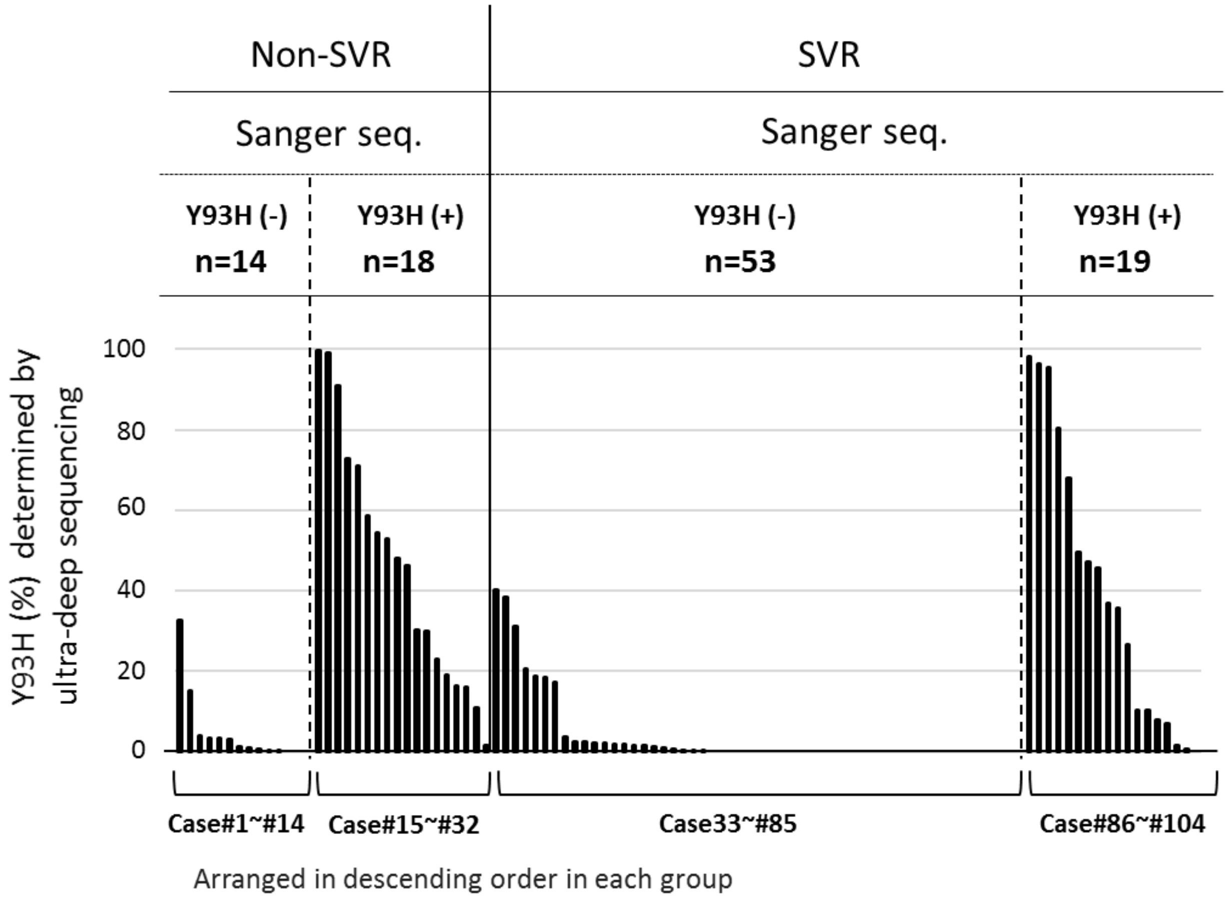

Supplementary Figure S2. Prevalence of Y93H-positive clones at baseline in SVR and non-SVR patients determined by ultra-deep sequencing analysis. Frequency of Y93H-positive clones determined by ultra-deep sequencing in non-SVR (left graph) and SVR (right graph) cases, and prevalence of Y93H determined by Sanger sequencing are shown according to treatment outcome.

Supplementary Figure S3

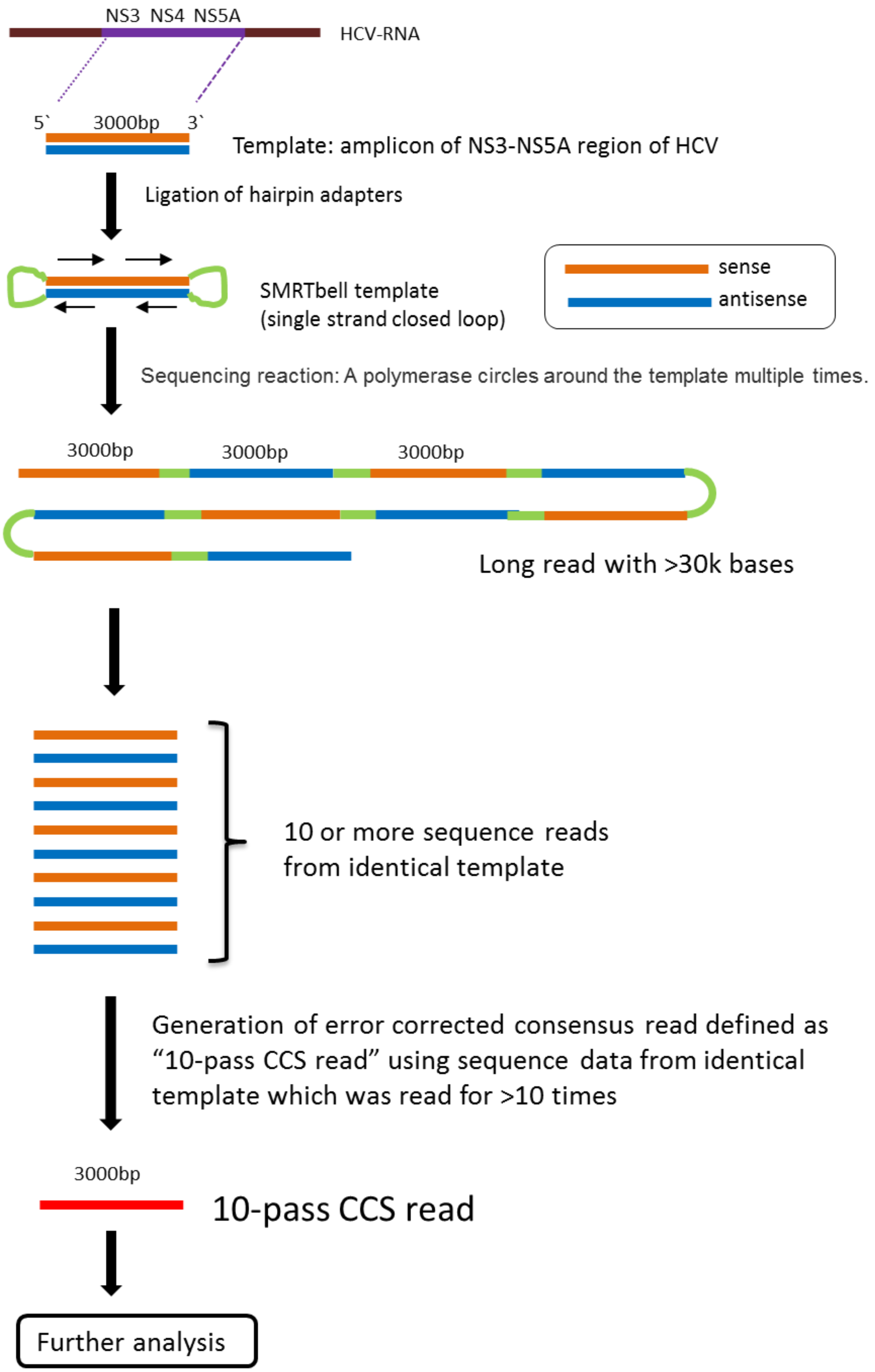

Supplementary Figure S3. Schemas showing the generation of circular consensus sequencing (CCS) reads. The template for PacBio sequencing, so-called SMRTBell, is created by ligating hairpin adaptors to both ends of double-stranded DNA molecules, and acts like a single-stranded closed circle. The enzyme starts sequencing at the primer location and sequences the template until it falls off. The enzyme goes around the hairpin on the other end of the SMRTBell, and can circle around multiple times. Then, error-corrected consensus reads, so-called CCS reads, are generated using the data from the identical template sequenced by multiple times. To obtain extremely high accuracy of each read, we strictly selected CCS reads making 10 or more passes around the closed loop SMRTbells; defined as "10-pass CCS reads".

Supplementary Figure S4

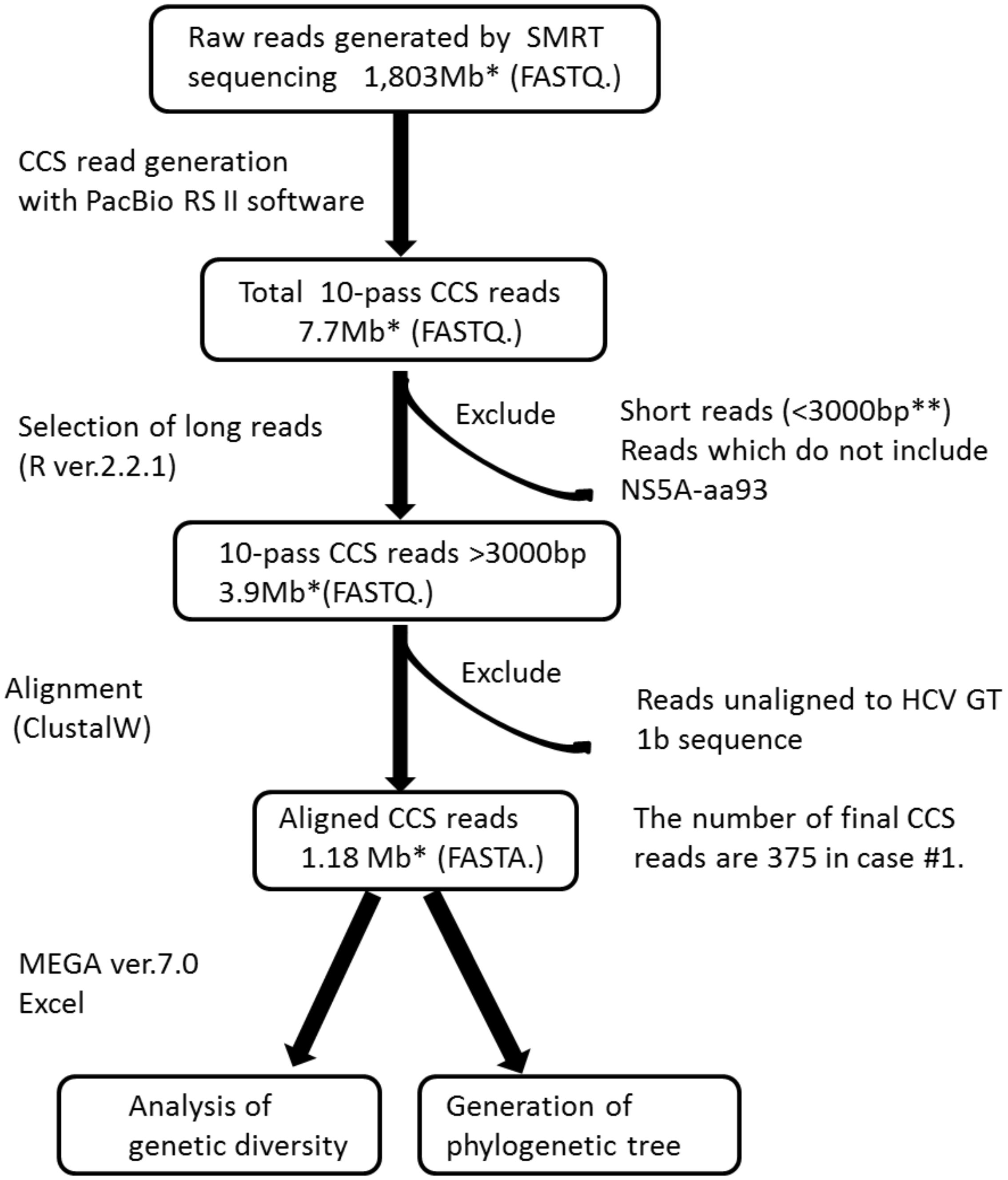

Supplementary Figure S4. Workflow for generation of CCS reads obtained by PacBio RSII sequencing platform. The representative file size of SMRT sequencing and generation of CCS reads are shown (Case#1 at baseline). From total raw reads generated by SMRT sequencing, 10-pass CCS reads are generated using PacBio RSII software. Using R ver.2.2.1 software, long reads (> 3000 bp) are selected. Sequence alignment was performed using ClustalW, and then the genetic diversity of HCV clones was analyzed and phylogenetic trees were generated using the maximum likelihood method.

Supplementary Figure S5

A

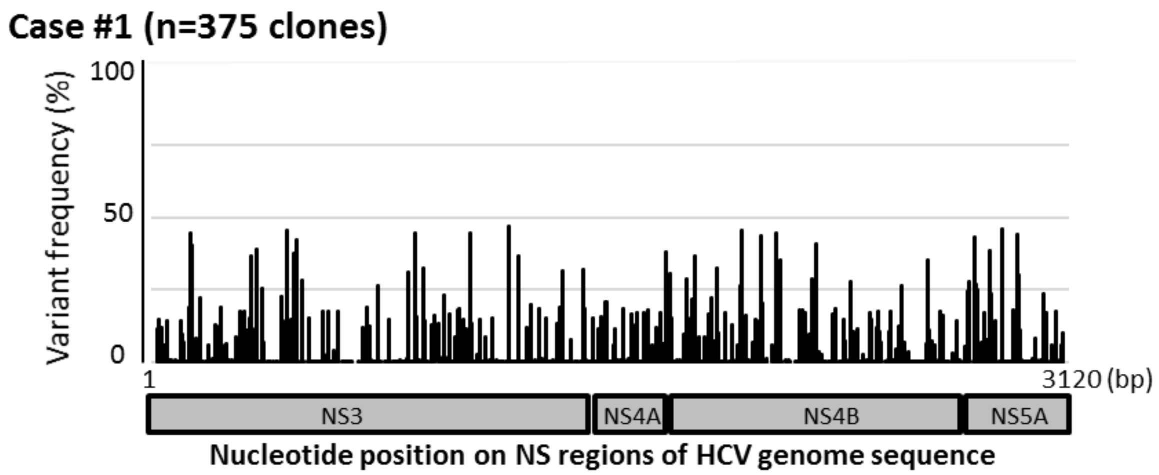

B

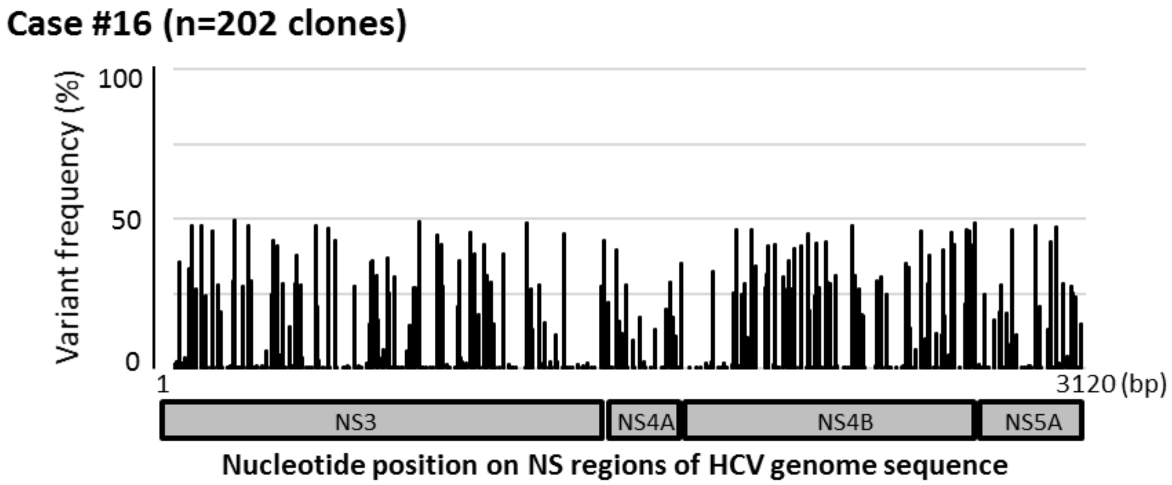

Supplementary Figure S5. Distribution of the nucleotide variant frequency in the NS region determined by third-generation sequencing platform. Variant frequencies at each nucleotide position in the NS region of two representative cases are demonstrated. Nucleotide 1 indicates the first nucleotide of the HCV template sequenced by third-generation sequencing platform. (**A)** Case#1, **(B)** Case#16.

Supplementary Table S1. Prevalence of resistant associated substitutions at pre- and post-DCV/ASV treatment in non-SVR patients.

Case #	Clinical characteristics					NS5A											NS3			
	Age and sex	LC or CH	HCV-RNA (pre)†	Previous DAA exposure	Reason for failure	Y93H			L31M/V/F/I		L28V/M		R30Q/K		Q54H		Q80R/K		D168V/M/E	
						Sanger	Deep		Deep		Deep		Deep		Deep		Deep		Deep	
						pre	Pre	Post	Pre	Post	Pre	Post	Pre	Post	Pre	Post	Pre	Post	Pre	Post
#1	61F	CH	6.6	TRV	VBT	-	3.0	99.8	-	92.7	-	-	-	-	-	-	-	-	-	99.7
#2	63F	LC	5.5	SMV	AE	-	1.4	30.1	-	-	-	-	-	20.2	8.7	68.6	98.5	54.0	-	99.8
#3	63F	CH	6.1	-	VBT	-	3.5	99.2	-	99.8	-	-	-	-	97.0	99.0	-	-	-	97.2
#4	78M	CH	5.4	-	VBT	-	-	96.6	-	95.0	-	-	-	98.1	-	-	-	30.6	95.5	88.0
#5	67M	LC	5.5	-	relapse	-	32.6	45.1	5.4	11.4	4.3	2.3	4.5	5.0	62.7	55.6	-	-	-	99.1
#6	63M	CH	6.9	SMV	NR	-	15.1	94.1	-	25.1	-	-	0.5	-	-	13.4	-	5.8	33.9	99.8
#7	67F	CH	3.6	-	VBT	-	4.1	50.0	8.4	16.0	8.1	26.9	8.2	30.2	73.6	31.2	-	-	46.6	99.6
#8	71F	LC	7.0	SMV	VBT	-	3.5	3.0	-	17.4	-	-	-	-	97.0	90.3	-	-	38.4	99.3
#9	68F	LC	6.5	-	relapse	-	1.0	21.9	-	31.8	-	-	-	-	68.5	54.4	-	-	-	31.8
#10	62M	LC	5.9	-	VBT	-	0.9	96.4	-	84.4	-	-	-	-	95.1	86.0	-	-	-	71.5
#11	73F	CH	6.3	-	relapse	-	0.7	99.5	-	98.0	97.2	-	-	-	-	-	-	0.9	2.2	99.0
#12	72F	LC	4.9	SMV	VBT	-	-	86.9	-	-	-	0.5	-	1.0	-	0.9	0.8	-	9.7	98.4
#13	72F	CH	6.4	SMV	VBT	-	-	-	-	88.5	-	-	13.4	6.0	66.6	20.9	-	-	95.5	99.3
#14	63F	LC	6.8	-	VBT	-	-	78.0	-	74.1	-	-	-	-	20.2	73.6	48.4	-	-	91.6
#15	63M	LC	6.3	SMV	NR	-	22.9	96.9	75.5	83.3	-	-	-	-	98.8	99.0	-	-	81.6	99.8
#16	75F	LC	6.6	-	AE	-	1.4	96.4	92.1	92.8	-	-	-	-	94.6	91.6	-	-	-	63.1
#17	55F	LC	7.1	-	VBT	+	90.8	99.4	98.5	98.9	-	-	-	-	-	-	-	-	-	40.7
#18	74F	CH	6.2	-	relapse	+	19.0	94.6	0.9	90.9	-	-	-	-	87.0	-	-	-	-	94.1
#19	63F	LC	6.5	-	relapse	+	99.6	NA	-	NA	-	NA	99.0	NA	72.7	NA	-	-	-	97.8

#20	76M	CH	6.5	-	relapse	+	10.8	31.8	-	5.0	-	-	-	8.3	-	93.6	98.8	96.3	-	93.9
#21	73F	LC	6.2	-	relapse	+	48.1	99.6	1.0	92.2	-	-	-	-	-	93.2	-	-	30.0	96.8
#22	64F	CH	6.2	-	relapse	+	16.3	-	-	95.0	-	4.8	-	-	98.9	99.1	-	NA	-	NA
#23	81F	LC	5.8	-	VBT	+	15.9	38.8	-	93.4	-	-	-	5.2	95.7	57.1	-	13.3	-	97.6
#24	63M	LC	5.9	-	VBT	+	99.2	NA	-	NA	-	NA	-	NA	-	NA	-	NA	-	NA
#25	77M	CH	6.4	-	relapse	+	30.0	NA	-	NA	-	NA	-	NA	98.0	NA	-	NA	-	NA
#26	72F	CH	6.1	-	AE	+	46.3	NA	13.4	NA	-	NA	8.1	NA	54.4	NA	-	NA	-	NA
#27	70F	CH	5.5	-	relapse	+	70.9	78.8	79.2	81.6	-	-	20.4	20.7	0.8	0.7	-	-	3.5	81.4
#28	64M	CH	7.3	SMV	NR	+	54.5	83.3	9.9	-	-	-	6.2	2.8	40.0	3.9	0.4	-	96.6	99.6
#29	65F	CH	6.6	-	relapse	+	58.5	63.7	3.0	-	1.4	0.6	2.0	0.6	58.1	38.9	0.5	-	-	-
#30	68M	CH	7.2	SMV	NR	+	52.8	32.3	-	35.6	-	-	-	-	-	-	-	-	16.0	60.8
#31	43M	CH	7.0	-	relapse	+	72.8	66.9	77.9	75.3	-	-	26.7	32.5	16.3	21.6	29.7	-	21.4	41.9
#32	77M	LC	6.7	-	VBT	+	30.1	55.7	-	45.5	-	1.0	28.2	-	21.3	49.0	-	-	-	99.8

M, male; F, female; LC, liver cirrhosis; CH, chronic hepatitis; SMV, simeprevir; TRV, telaprevir; VBT, viral breakthrough; NR, non-responder; AE, adverse event, †, LogIU/mL; ‡, months after the initiation of treatment; pre, prior to treatment; post, after treatment failure, NA, not available; -, not detectable

Supplementary Table S2. Error rate of SMRT platform calculated from the control study with HCV-plasmid

Variable	
A total bases of generated CCS reads (A)	2,320,591 (bp)
Matched bases	2,312,463 (bp)
Mismatches (B)	641 (bp)
Deletions (C)	7,316 (bps)
Insertions (D)	171 (bp)
Mismatch error rate (B / A)	0.000287
Total error rate (B+C+D / A)	0.0035

SMRT, single molecular real-time; CCS, circular consensus sequence

Supplementary Table S3. Pretreatment viral diversity demonstrated by SMRT sequencing.

Case #	Read length analyzed (bps) (A)	Number of CCS reads analyzed	Number of nucleotide changes of each CCS read compared with each consensus sequence					Average variant frequency of each CCS read (= B/A)
			Mean (B)	SD	95%CI	Median	Range	
#1	3120	375	73.2	8.7	72.3-74	75	46-95	0.023461538
#17	3120	210	18.2	20.4	15.4-21.0	10	3-111	0.005833333
#4	3120	197	97.6	31.7	93.4-101.9	101	37-178	0.031282051
#2	3120	508	37.3	22	35.3-39.2	31	9-161	0.011955128
#16	3120	196	58.9	7.6	57.8-56.0	59	39-81	0.018878205
#3	2074	284	75.7	9.4	74.6-76.8	76	46-105	0.036499518

Abbreviations: SMRT, single molecule real-time; CCS, circular consensus sequence; SD, standard deviation; CI, confidence interval

Supplementary Table S4. Oligonucleotides used as PCR primers for sequencing of NS3 or NS5A regions

Primers	Sequence	Base position*	Purpose
Fw1	TCCAGCCTTACCATCACTCA	5845-5864	RT-PCR for NS5A region
Fw2	TGAAGAGGCTTCATCAGTGG	5964-5982	nested PCR for NS5A region
Rv1	CCCGTCACGTAGTGGAAATC	6311-6292	RT-PCR for NS5A region
Rv2	GCGTAACCTCCACGTACTCC	6283-6264	nested PCR for NS5A region
Fw3	ACAGGTCGGGACAAGAACCAG	3142-3162	RT-PCR for NS3 region
Fw4	ACAAGAACCAGGTCGATGGGGAG	3152-3174	nested PCR for NS3 region
Rv3	GAGACCGCATGGTAGTTTCCAT	3622-3601	RT-PCR for NS3 region
Rv4	GGTAGTTTCCATAGACTCAACGG	3612-3590	nested PCR for NS3 region

*: counted from the start codon of Core region. RT; reverse transcription, PCR; polymerase chain reaction
